# Aortic Root Dilatation With Healed Aortic Root Abscess, Asymptomatic for 41 Years

**DOI:** 10.7759/cureus.10113

**Published:** 2020-08-29

**Authors:** Akshit Chitkara, Anamika ., Varun Kumar Sharma, Piyush Puri, Sneha Pusapati

**Affiliations:** 1 Internal Medicine, Sh. Moolchand Super Speciality Hospital, Karnal, IND; 2 Internal Medicine, Mahatama Gandhi Institute of Medical Sciences, Hisar, IND; 3 Internal Medicine, Government Medical College, Amritsar, IND; 4 Internal Medicine, Al Flah School of Medical Sciences and Research Center, Faridabad, IND; 5 Internal Medicine, State University of New York (SUNY) Downstate, New York, USA

**Keywords:** aortic root dilatation, aortic root abscess

## Abstract

A 61-year-old patient presented to us with complaints of mild fever, rhinorrhea, sneezing, and dry cough. On auscultation, there was harsh vesicular breath sound with bilateral audible wheezing but no crackles. Also, a soft high-pitched early diastolic decrescendo murmur in third intercostal space on the left side, typical of aortic regurgitation (AR) was heard. History revealed that in 1976 he was diagnosed with AR and later in 1990 he was diagnosed with aortic root dilatation (AoD) with healed aortic root abscess (ARA). He had no history of infective endocarditis and has remained asymptomatic. The latest echocardiography revealed mild left ventricular hypertrophy (LVH) with Grade 1 LV diastolic dysfunction but no left ventricular (LV) wall motion abnormality. The patient was advised to take tablet losartan 20 mg OD to arrest the progression of his mild LVH and its related complications and continue with his lifestyle management and routine echocardiography.

In some rare cases, AoD with healed ARA can remain asymptomatic over the course of decades. Regular follow up exams (every one to two years), with proper management, is the mainstay of management, along with the treatment of comorbid conditions.

## Introduction

This report describes the management of a case with a rare combination of aortic root dilatation (AoD) and healed aortic root abscess (ARA) that remained asymptomatic for over 40 years. There are significant morbidity and mortality rates associated with these structural heart diseases, and proper management plans and timely intervention are paramount to managing such cases [1].

## Case presentation

A fit-and-well 61-year-old male presented to us with complaints of mild fever, rhinorrhea, sneezing, and dry cough for the past five days. He had normal bowel and urinary functions. There were no complaints of chest pain or hemoptysis. He had no past medical history of diabetes mellitus, obesity, or hypertension. He had no smoking or drinking habits. Also, no family history of diabetes mellitus, hypertension, or coronary artery disease. In his family history, both the parents had bronchial asthma. Physical examination revealed no edema on feet, cyanosis, or pallor. Body mass index (BMI) was 25 and jugular venous pressure (JVP) was normal, with no lymphadenopathy and no hepatosplenomegaly. The throat was slightly congested. On auscultation, there was harsh vesicular breath sound with bilateral wheezing but no crackles. A soft high-pitched early diastolic decrescendo murmur in third intercostal space on the left side typical of aortic regurgitation (AR) is heard. He had a heart rate of 88 beats per minute and the pulse was bounding and regular, respiratory rate of 20 breaths per minute, blood pressure of 122/78 mmHg, and temperature of 98.6°F.

**Figure 1:**
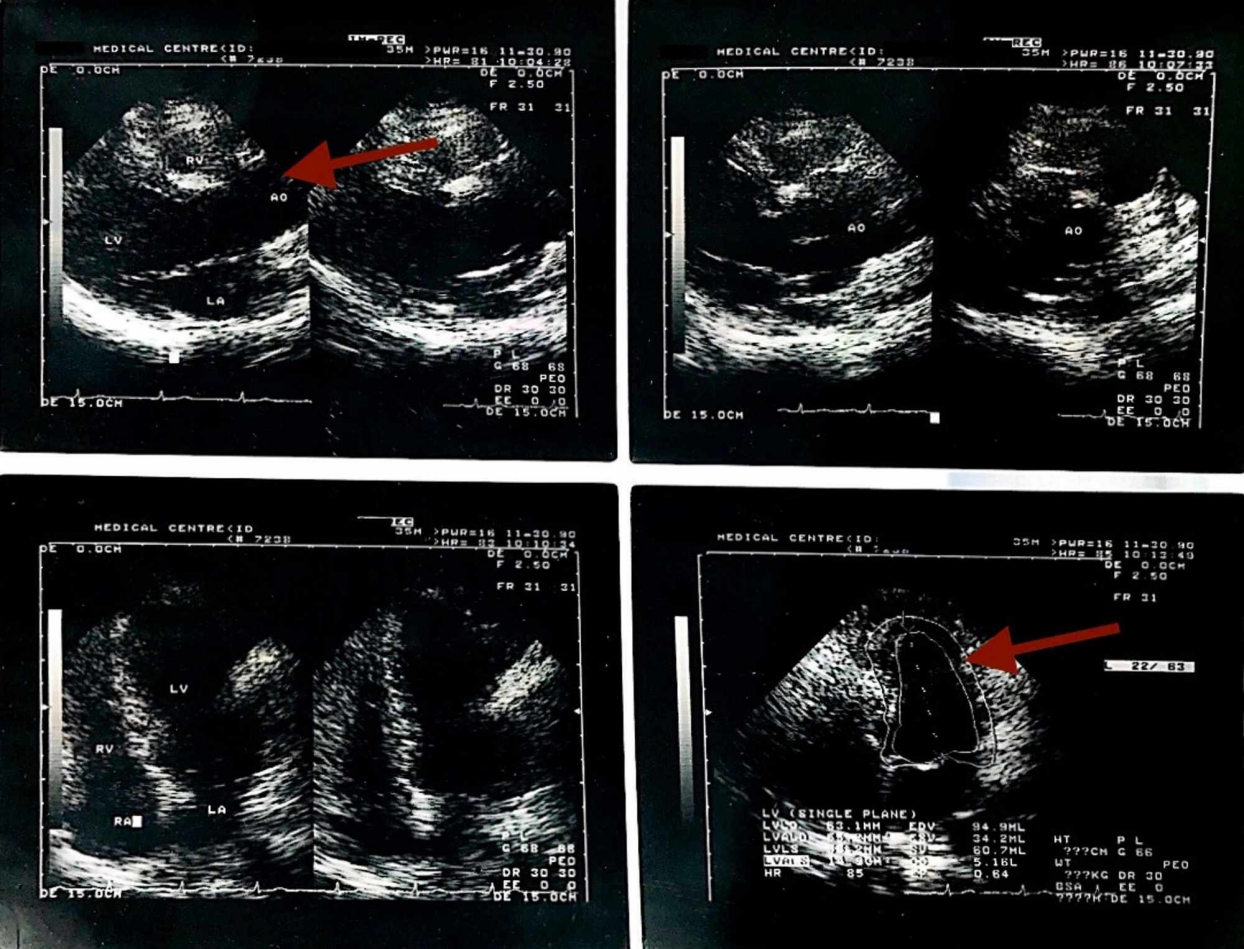
Transthoracic echocardiographic images of the heart - Nov 1990.

**Figure 2:**
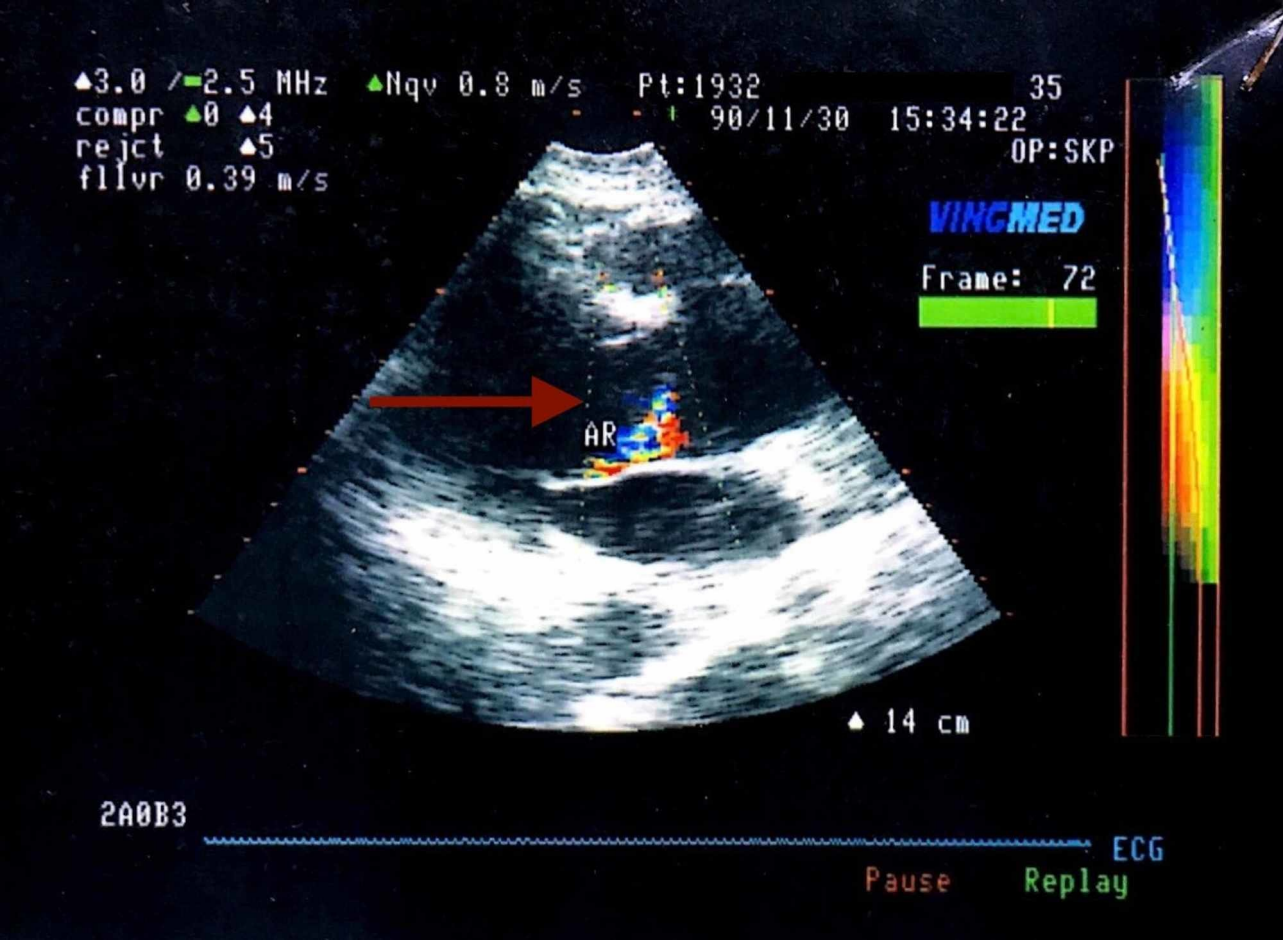
Showing AR into the LV - Nov 1990. AR, aortic regurgitation; LV, left ventricle

On further interrogation, the patient told that when he was 20 years old (41 years ago), he was informed of a mild AR on auscultation by his family physician during a routine health exam, though he was totally asymptomatic at that time. Further investigations were not possible due to the unavailability of echocardiography in India in the 1970s. As echocardiography became available around 30 years ago, he consulted two cardiologists and one radiologist in the year 1990, for a complete cardiac assessment with echocardiography, though he was still asymptomatic. From the echocardiography, it was revealed that he had an AoD (Figure 1) (diameter 45 mm and 49 mm) with a mild AR (Figure 2), and mild enlargement of left ventricle (LV) but no evidence of LV hypertrophy (LVH).

Right ventricular and LV functions were good with an LV ejection fraction of 64%.

**Figure 3:**
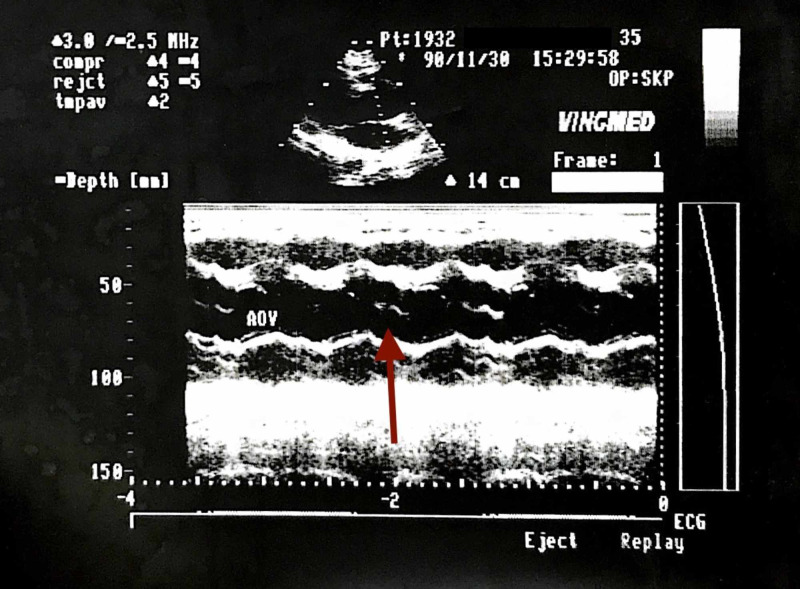
Blood flow is seen in the channel posterior to the membrane - Nov 1990.

There was an anterior mitral leaflet (AML) flutter seen due to an AR jet hitting the AML. There was no ventricular wall motion abnormality. There was a linear echo dense structure along the sinus of the noncoronary cusp; this moved parallel to the aortic wall and did not show any flutter, and an echo-free space was seen below the noncoronary cusps which disappeared with slight angulation of the transducer. The flow could be seen in the channel posterior to the membrane mentioned above (Figure 3), no entry/exit points could be identified.

Suprasternal and right parasternal views did not show any aortic dissection or any false lumen. The consensus of the cardiologists and the radiologist, back in 1990, was that the small channel seen in relation to the noncoronary cusp (NCC) of the aortic valve could be a healed ARA with AoD, though there was no history of endocarditis at any stage. Subsequent echocardiography every two years did not show any worsening of the condition.

**Investigations**

**Figure 4:**
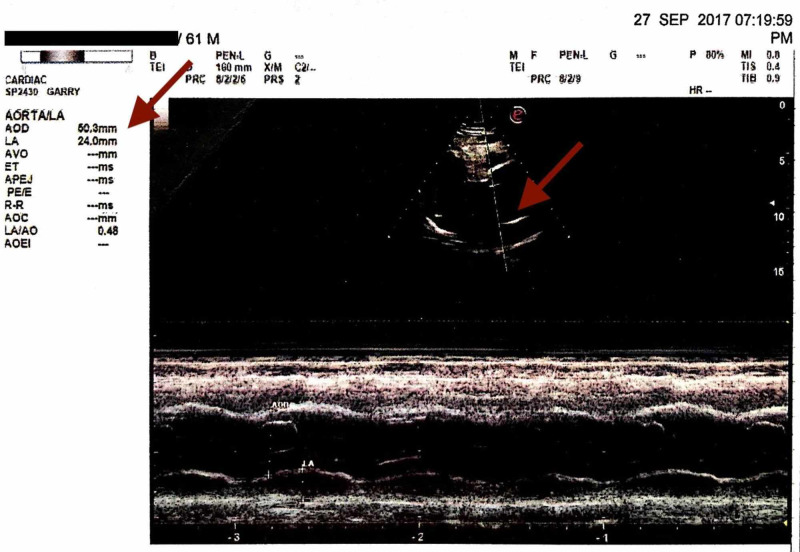
Measurement of the aortic root diameter - 50.3 mm - Sep 2017.

**Figure 5:**
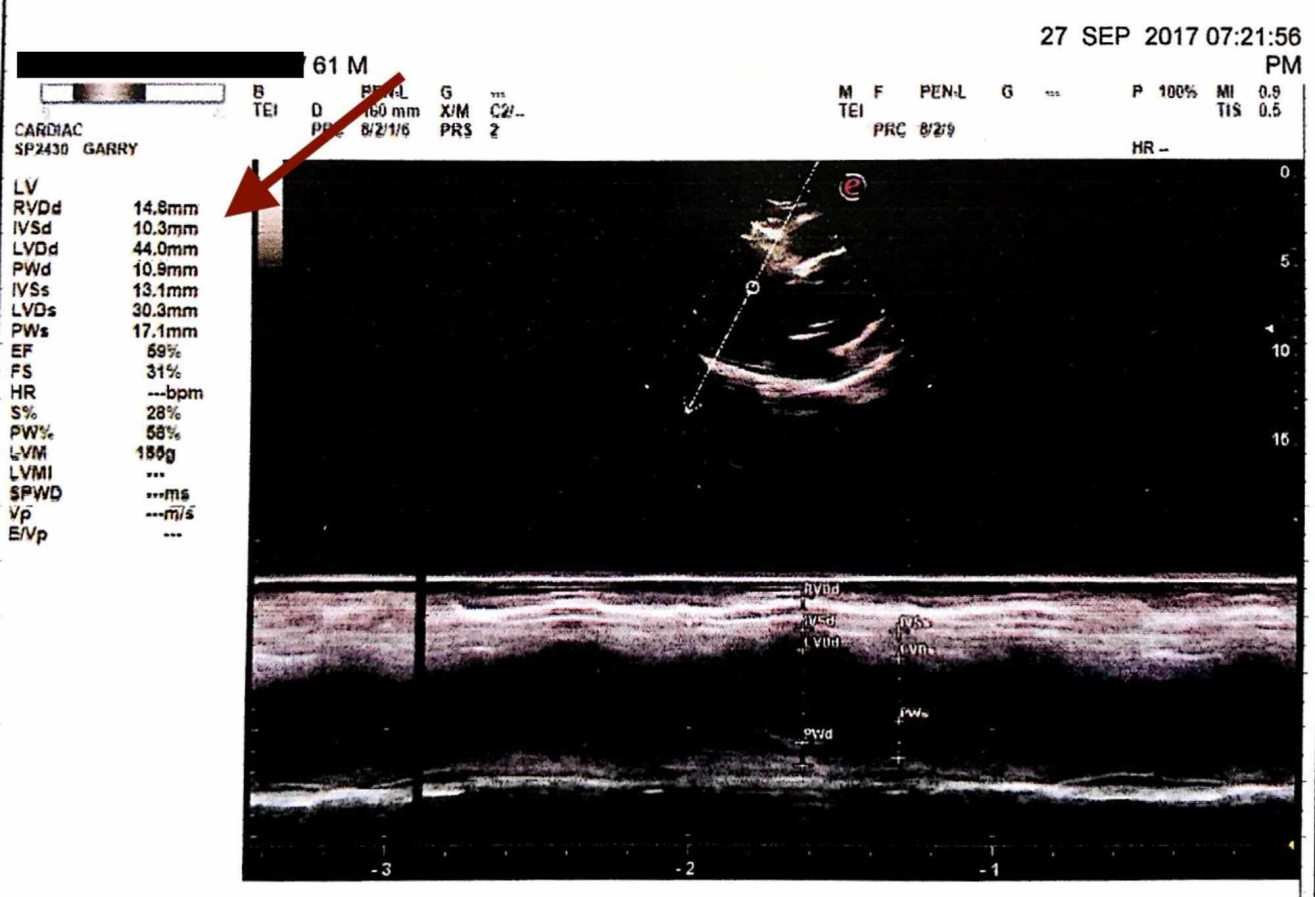
Mild LVH - Sep 2017. LVH, left ventricular hypertrophy

Venereal disease research laboratory (VDRL) of nonreactive, X-Ray chest within normal limits (WNL), electrocardiogram (ECG) - WNL, X-Ray bilateral hands - normal (to rule out Marfan's syndrome). The latest echocardiography revealed an aortic root diameter of 50.3 mm (Figure 4), ejection fraction is 60%, mild LVH (Figure 5) with Grade 1 LV diastolic dysfunction but no LV wall motion abnormality. LV end-systolic pressure of 30.3 mmHg, LV end-diastolic pressure of 44 mm Hg. The right ventricle, right atrium, and left atrium are normal. Pulmonary valve, tricuspid valve, mitral valve, and AML are normal.

**Treatment**

In order to arrest the progression of his LVH, the patient should be prescribed either beta-blockers, ACE inhibitors, angiotensin II receptor blockers (ARBs), or calcium channel blockers (CCBs), and to continue with his lifestyle management and routine echocardiography exams. Upon consultation with another cardiologist, we advised him to take tablet losartan 20 mg OD to arrest the progression of his mild LVH and its related complications.

## Discussion

Aortic root abscess is a very serious disease with multiple inherent complications [2]. AoD is most commonly an asymptomatic condition usually discovered on routine echocardiography. AoD in some cases may complicate in the form of AR, LV dilatation, LVH, aortic dissection, aortic aneurysm, or aortic rupture. ARA most commonly occurs as a complication of infective endocarditis and is more frequently seen in patients of prosthetic aortic valves [3]. It is accompanied by much higher morbidity and mortality. Transesophageal echocardiography is the most preferred imaging technique for its diagnosis [4]. ARA may erode the surrounding tissues and lead to cardiac fistula, aortic dissection, aortic aneurysm, or aortic rupture. Staphylococcus aureus is the most common organism in ARA/infective endocarditis. The treatment involves medical as well as surgical intervention and the surgery itself is accompanied by significant morbidity and mortality [2].

In our patient, both AoD and healed ARA coexisted, and he has been leading a normal and active life for the last four decades. There is no past history of Syphilis, Marfan’s and Ehlers-Danlos syndrome in his medical records, and no other congenital abnormality. He never had any symptoms related to this double cardiac anomaly. The diameter of the aortic root and the healed ARA have remained unchanged in spite of his busy and active lifestyle during this period of 30 years.

The LVEF has remained almost the same (more than 60%), but there is mild LVH which has not led to the development of any symptoms so far. The main contributing factors to his remaining asymptomatic were probably him being a nonsmoker and not have any comorbid conditions such as diabetes mellitus, hypertension, or obesity, along with a healthy diet and an active lifestyle [5]. In order to arrest the progression of his LVH, the patient should be prescribed either beta-blockers, ACE inhibitors, ARBs, or CCBs, and to continue with his lifestyle management and routine echocardiography [6].

## Conclusions

In some rare cases, as we saw in our patient, AoD with healed ARA can remain asymptomatic over the course of decades. In such patients, regular follow up exams (every one to two years), with echocardiography, is the mainstay of management, along with the treatment of comorbid conditions such as diabetes mellitus, hypertension, obesity, and coronary artery disease. Coexisting heart conditions such as AoD with a healed or an active ARA require expertise on the part of treating physician, because both of these ailments can progress over time, and may lead to serious and life-threatening conditions such as AR, LV dilatation, LVH, aortic dissection, aortic aneurysm, or aortic rupture.
